# Spinal hydatidosis mimicking Guillain Barre syndrome: in case of doubt there is no rush to perform lumbar puncture

**DOI:** 10.11604/pamj.2014.19.348.5783

**Published:** 2014-12-03

**Authors:** Ahmed Belkouch, Abdelilah Mouhsine, Rachid Sirbou, Saad Zidouh, Taoufik Bakkali, Abdelghani El Fikri, Lahcen Belyamani

**Affiliations:** 1 Emergency Department, Mohammed V Military Hospital of Instruction, Faculty of Medicine and Pharmacy, Rabat, Morocco; 2 Department of Radiology, Avicenna Military Hospital, Faculty of Medicine and Pharmacy, Marrakech, Morocco

**Keywords:** Guillain Barre Syndrome, lumbar puncture, spinal hydatidosis

## Abstract

Guillain Barre Syndrome (GBS) is a challenging pathology which diagnosis is based essentially on the clinical examination and the results of lumbar puncture. Differential diagnosis must be discussed if the clinical picture is not complete. We present the case of a patient who presented to the emergency department with symptoms evoking both GBS and spinal cord compression. The Radiology showed a diffused spinal hydatidosis. The lumbar puncture must be carefully considered. In this case, it would have exposed the patient to hydatid dissemination.

## Introduction

Guillain Barre Syndrome is a part of the neurological pathology seen essentially in the emergency department. First described in France as a cause of acute flaccid paralysis, it was distinguished from poliomyelitis by the albuminocytological dissociation found in the cerebro-spinal fluid [1]. The clinicalexaminationis the key to the diagnosis. Symptoms on the oncet are dominated by the motor neurological deficit and tendon areflexia.

## Patient and observation

**Figure 1:**
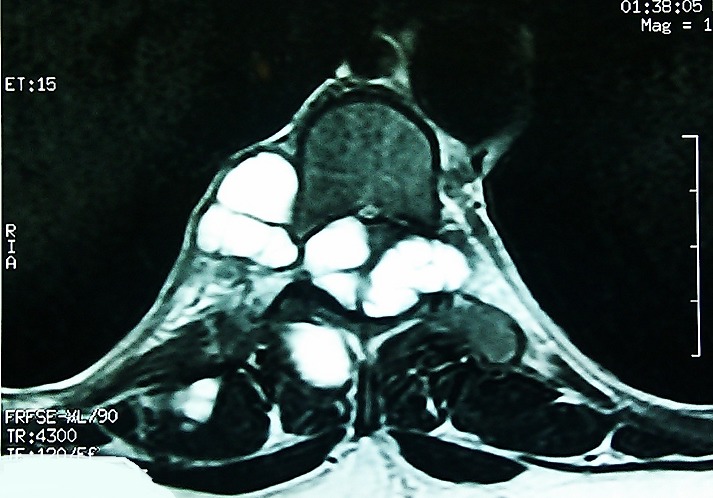
MRI in transversal view showing spinal cord compression by the vesicles of hydatid cysts

**Figure 2:**
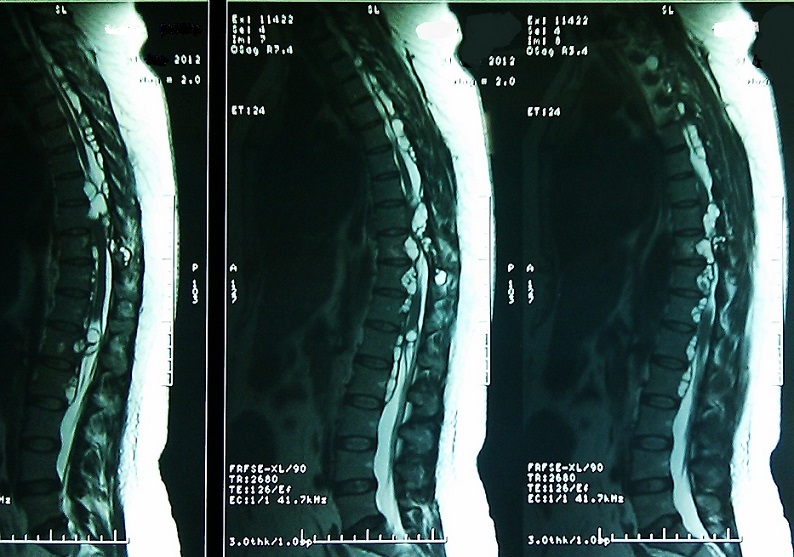
Diffused spinal hydatidosis in the sagittal and parasagittal views

A thirty-six years old woman, with no medical history, was admitted to the emergency room for a para paresis that developed gradually. The onset of symptoms dated back to 2 weeks by a tingling sensation in her toes, foot and after that in her legs with gradual onset of numbness making walking impossible. The patient noted no spinal pain or stiffness or sphincter dysfunction. She did not describe infectious syndrome in the days preceding the neurological symptoms. Clinical examination showed no loss of consciousness, the patient was apyretic, blood pressure and heart rate were normal, the respiratory rate was about 15 cycles/min. neurological examination showed a flaccid para paresis, tendon reflexes were diminished on both sides. The neck was supple and there was no deformation of the spine. The cranial nerves examination was normal, and the patient was unable to walk with greater right foot drop. The rest of the clinical examination was without abnormalities. A GBS was suspected but the clinical symptomatology was not complete. We also discussed a spinal cord compression. Since the patient was stable we preferred to perform first MRI that found a large number of cysts in the spinal cord (Figure 1, Figure 2). As we are an endemic country of hydatidosis we suspected that pathology. It was confirmed by surgery and we also discovered other hydatid cysts in the liver.

## Discussion

The Guillain-Barré syndrome is a rare disease with an incidence of 1.5 per 100 000 people [2]. Described for the first time in 1916, the diagnosis is based on clinical presentation and albuminocytologic dissociation [1]. Since then, scientific knowledge evolved and several subtypes have been distinguished. The most frequent is the acute inflammatory demyelinating polyradiculoneuropathy (AIDP). The illness develops in four phases [3]: the prodromal phase, the phase of expansion of the paralysis, the plateau phase and finally the recovering phase. In the Emergency department, only the two first phases are observed. The onset is usually marked by a respiratory infection syndrome (Mycoplasma pneumoniae) or a digestive infection (Campylobacter jejuni). Then the extension phase of paralysis occures few days after. The motor neurological deficit is ascending, usually symmetrical, begins with the lower limbs, sometimes to the four members and has a gradual and steady progress towards the root of the member [3].

The clinical criteria required for diagnosis are [4] progressive weakness in both arms and legs (might start with weakness only in the legs) and areflexia (or decreased tendon reflexes). Features that strongly support the diagnosis [4]: progression of symptoms over days to 4 weeks; relative symmetry of symptoms; mildsensorysymptoms or signs; cranial nerve involvement, especially bilateral weakness of facial muscles; autonomic dysfunction; pain (often present); high concentration of protein in CSF; typical electrodiagnostic features. In typical cases there is pain, numbness, paresthesia or weakness in the limbs [4]. The great clinical diversity makes diagnosis difficult, and other diagnoses may be discussed in approximately 10-15% of cases [4]. The GBS treatment is an emergency which aims to limit the extension of motor deficit [4].

In our case the patient had all symptoms required to evoke the diagnosis of GBS. However, the lack of an infectious syndrome made us think of a spinal cord compression, so we decided to perform imaging before. Indeed, a lumbar puncture would have exposed our patient to a breaking of a hydatic cyst in the peri arachnoid spaces.

## Conclusion

The Guillain Barre Syndrome is a challenging condition because of differential diagnosis with spinal cord compression. The emergency physician needs to know when to evoke the diagnosis and the lumbar puncture should be reflected. In case of doubt imagery must be performed first.
